# Parent Rated Symptoms of Inattention in Childhood Predict High School Academic Achievement Across Two Culturally and Diagnostically Diverse Samples

**DOI:** 10.3389/fpsyg.2017.01436

**Published:** 2017-08-25

**Authors:** Astri J. Lundervold, Jocelyn I. Meza, Mari Hysing, Stephen P. Hinshaw

**Affiliations:** ^1^Department of Biological and Medical Psychology, University of Bergen Bergen, Norway; ^2^K. G. Jebsen Center for Neuropsychiatric Disorders, University of Bergen Bergen, Norway; ^3^Department of Psychology, University of California, Berkeley, Berkeley CA, United States; ^4^Regional Centre for Child and Youth Mental Health and Child Welfare, Uni Research Health Bergen, Norway; ^5^Department of Psychiatry, University of California, San Francisco, San Francisco CA, United States

**Keywords:** childhood inattention, academic achievement, culturally diverse, intellectual functioning, Bergen Child Study, Berkeley Girls with ADHD Longitudinal Study

## Abstract

**Objective:** To investigate parent reports of childhood symptoms of inattention as a predictor of adolescent academic achievement, taking into account the impact of the child’s intellectual functioning, in two diagnostically and culturally diverse samples.

**Method:** Samples: (a) an all-female sample in the U.S. predominated by youth with ADHD (Berkeley Girls with ADHD Longitudinal Study [BGALS], *N* = 202), and (b) a mixed-sex sample recruited from a Norwegian population-based sample (the Bergen Child Study [BCS], *N* = 93). Inattention and intellectual function were assessed via the same measures in the two samples; academic achievement scores during and beyond high school and demographic covariates were country-specific.

**Results:** Childhood inattention predicted subsequent academic achievement in both samples, with a somewhat stronger effect in the BGALS sample, which included a large subgroup of children with ADHD. Intellectual function was another strong predictor, but the effect of early inattention remained statistically significant in both samples when intellectual function was covaried.

**Conclusion:** The effect of early indicators of inattention on future academic success was robust across the two samples. These results support the use of remediation procedures broadly applied. Future longitudinal multicenter studies with pre-planned common inclusion criteria should be performed to increase our understanding of the importance of inattention in primary school children for concurrent and prospective functioning.

## Introduction

Academic achievement is an important predictor of later vocational career success and adult financial stability (Richardson et al., 2012; Fried et al., 2016). Identification of predictors of academic success is therefore of great importance. The link between externalizing behavior and academic underachievement is well-documented (Hinshaw, 1992a), particularly in children with attention-deficit/hyperactivity disorder (ADHD) (Hinshaw, 1992b; Loe and Feldman, 2007; Spencer et al., 2007; Daley and Birchwood, 2010; Owens and Jackson, 2017). Other studies have emphasized the importance of inattention, one of the core symptom domains of ADHD. This association is illustrated by longitudinal follow-up studies of girls participating in the Berkeley Girls with ADHD Longitudinal Study (BGALS; see Lee and Hinshaw, 2006; Owens et al., 2017) and substantiated by Garner et al. (2013). In the latter investigation, based on teacher and parent ratings of inattention and hyperactivity-impulsivity symptoms in a large sample of 5663 boys and girls with ADHD (age range: 3–17), teacher ratings of inattention emerged as the strongest predictor of later academic achievement.

Symptoms of inattention, however, are not restricted to a specific diagnostic group. Most patients with neuropsychiatric disorders (e.g., Carmichael et al., 2015), as well as a proportion of the general population (e.g., Polderman et al., 2007), display some degree of problems related to inattention. In a systematic review of prospective studies, Polderman et al. (2010) confirmed that inattention is a core risk factor for poor academic achievement in both clinical and population-based samples. Pingault et al. (2011) performed an in-depth study of developmental trajectories in a 16-year longitudinal study of a French-Canadian community sample. They found that the risk of graduation failure dramatically increased in boys and girls who displayed rising rates of inattention during the period from childhood through adolescence. A population-based Swedish study by Holmberg and Bolte (2014) showed that a high risk for academic failure in upper secondary school (16 years of age) could be directly predicted from teacher-reported problems related to inattention in primary school (7 years of age). A direct effect of inattention to lowered academic achievement was also convincingly shown in a longitudinal study including a community sample of elementary school children aged 5–7 years (Gray et al., 2015). Furthermore, a recent investigation showed that the association between inattentive behavior in kindergarten and academic function in eighth grade was strong among children with both severe and less severe ADHD (Owens and Jackson, 2017). Taken together, these findings suggest that inattention in early childhood predicts later academic problems across diverse samples. Results from different studies are, however, difficult to compare because of cultural as well as methodological differences. Through the present investigation we aim to add to this literature by including ratings of inattention and later academic achievement in two culturally and diagnostically diverse samples—yet with key methodological similarities, which afford direct comparison of findings.

We note that most previous studies in this area feature teacher ratings of inattention, often to the exclusion of parent ratings of this variable (e.g., Pingault et al., 2011; Rogers et al., 2011). In addition, reflecting longstanding trends across all of child and adolescent psychopathology (Achenbach et al., 1987), agreement between teacher and parent ratings of problem behavior, including inattention, is low in both ADHD (Mitsis et al., 2000; Murray et al., 2007) and typically developing samples (Mares et al., 2007). A possible explanation is that teachers and parents may rate different aspects of inattention that occur in different environments (i.e., in school vs. at home). Because less is known about how parent’s ratings of inattention predict later academic achievement, we feature this information source in the present report.

Items used to assess inattention are closely related to cognitive/executive functions like flexibility, inhibition, and working memory, and are thus related to broader issues of executive function, as articulated in the classic review of Barkley (1997). This pattern suggests that a direct effect of inattention on academic achievement may be mediated or explained by key aspects of cognitive function. For example, Gray et al. (2015) found that the direct effect of early inattention on later academic performance was partially mediated by a test of working memory. Given that aspects of dysfunctional cognition are described as a core component of ADHD (e.g., Sonuga-Barke, 2005; Barkley et al., 2008), an additive effect of inattention and cognitive dysfunction may be expected within this diagnostic group. Indeed, in adults with ADHD, those with co-existent cognitive dysfunction obtain lower academic success than those without (Biederman et al., 2006; Halleland et al., 2015). Together, these studies emphasize the importance of taking cognitive function into account when predicting academic achievement from early inattention symptoms.

Selection of cognitive measures is, however, challenging. Cognitive function may be defined from a single test or clusters of tests designed to assess the parallel cognitive domains or subfunctions (see, e.g., Miyake et al., 2000; Salthouse et al., 2003). It may also be defined as an overall “g” (or general) factor. Arguments for the latter are based on the moderate to strong correlation across tests of different cognitive processes. Intellectual function represents such an overall g-factor, revealing strong predictive associations to academic performance (Deary, 2012). We therefore include intellectual function as a proxy for overall cognitive function in this report.

### Current Study

We investigate the relation between parent-rated inattention in primary school children and their future academic success in high school by including the following two culturally and diagnostically diverse samples: (a) the Berkeley Girls with ADHD Longitudinal Study (BGALS), comprising girls with an ADHD diagnosis and a matched comparison group who attend or have attended high school (see Hinshaw, 2002; Hinshaw et al., 2012); and (b) a sample recruited from the population-based Bergen Child Study (BCS, Heiervang et al., 2007), including high-school youth. Based on analyses of these two datasets, we investigate the strength of the inattention-academic achievement link in the two samples, as well as the influence of intellectual function on this link.

In both samples, parents rated their children’s inattentive behavior according to the nine inattention items from the Swanson, Nolan, and Pelham Questionnaire (SNAP-IV, Swanson, 1992). Intellectual function was assessed via the third edition of the Wechsler Intelligence Scale for Children (WISC-III; Wechsler, 1991). Information about academic achievement was assessed during adolescence or early adulthood according to national standards. Both the BGALS and BCS leverage a prospective longitudinal design, with information from two study waves being examined for the present paper: a wave in childhood and an approximately 10-year follow-up wave, at the end of adolescence. From previous studies, reviewed above, we expect to find a strong link between parent reports of inattention and future academic achievement in both samples, with the strongest effect of inattention in the BGALS, because this sample has a majority of youth with a formal ADHD diagnosis. A close relation between intellectual function and parent reports of inattention is also expected to be stronger in the BGALS than in the BCS sample. We predict that adjusting for childhood IQ will reduce the predictive effect of inattention on later academic performance to a non-significant level in the BGALS – because of its large ADHD subsample – but that inattention will remain as a significant predictor in the BCS sample, which is drawn from the general population.

## Materials and Methods

### Sample 1: Berkeley Girls with ADHD Longitudinal Study

Girls aged 6–12 (born 1984–1992) were recruited from schools, mental health centers, pediatric practices, and direct advertisements to participate in summer research programs in the San Francisco Bay Area, California, United States in 1997, 1998, and 1999. After initial screening, participants with ADHD were included at Wave 1 (ages 6–12 years, mean = 9.6, *SD* = 1.7) if they met full ADHD diagnostic criteria for the combined or inattentive types on the parent-administered Diagnostic Interview Schedule for Children (DISC-IV; Shaffer et al., 2000). Disorders that commonly co-occur with ADHD (i.e., oppositional defiant disorder, conduct disorder, anxiety disorders, depression, and learning disorders) were allowed to enhance the generalizability of the sample. Comparison participants were matched to the ADHD sample on age and ethnicity but could not meet diagnostic criteria for ADHD on parent ratings or structured clinical interview. Exclusionary criteria for both groups were intellectual disability, pervasive developmental disorders, psychosis or overt neurological disorder, non-English spoken in the home, and a medical problem prohibiting summer camp participation.

Hundred and forty girls with and 88 age- and ethnicity-matched comparison girls without ADHD were selected after extensive screening (including the SNAP-IV; Swanson, 1992) and full diagnostic assessments (Hinshaw, 2002), including a test of intellectual function. Participants were invited to take part in prospective follow-up assessments 5 years later (age 11–18 years, mean = 14.2, *SD* = 1.6; see Hinshaw et al., 2006) and then 10 years later (age 17–24 years, mean = 19.6, *SD* = 1.7; see Hinshaw et al., 2012). Information about late-adolescent academic achievement at the 10-year follow-up is included in the present study. Aided by the use of social media in some cases, we located 216 of the 228 participants from the initial study-wave (95%). Based on 23 statistical comparisons to the retained sample, the12 participants lost to follow-up had lower family incomes and full-scale IQ scores and higher teacher-rated ADHD, externalizing, and internalizing symptoms, suggesting that the group lost to follow-up was more cognitively and behaviorally impaired.

The present sample includes girls with parent-reported information during Wave 1 on the inattention measure as well as performance on a well-standardized test of intellectual function. The measures of academic performance were obtained during Wave 3. The final sample comprised 202 children with complete data, including 128 with ADHD. All assessment waves received full approval from the institution’s Committee for the Protection of Human Subjects.

### Sample 2: Bergen Child Study

The BCS is a longitudinal, population-based study on mental health and development, including three age cohorts of children born between 1993 and 1995. When launched in 2002, it included all children attending any school in the city of Bergen, the second largest city of Norway. During the initial screening phase, a four-page BCS questionnaire was given to both the parents and teachers of the target population, including, among other scales, a slightly modified SNAP-IV (Swanson, 1992). In a second phase of the first study wave, a subset of parents was interviewed using the Development and Well-Being Assessment (DAWBA) (Goodman et al., 2000). All children who obtained any diagnosis according to DAWBA and an equal subgroup of children without a diagnosis were invited to participate together with their parents in a thorough clinical examination, designed to resemble a clinical psychiatric examination. A total of 329 children participated in this clinical study, which included a diagnostic interview: the Schedule of Affective Disorders and Schizophrenia for School-Age Children-Present and Lifetime version (K-SADS-PL) (Kaufman et al., 1997), plus the WISC-III (Wechsler, 1991). A fourth wave of the study invited a more comprehensive sample of all adolescents born between 1993 and 1995 living in the county of Hordaland, which includes the city of Bergen. The BCS sample was thus nested within this young@hordaland sample.

The sample for the present study includes participants with parent reports on inattentive behavior from the initial study wave (mean age = 8.14, *SD* = 0.81), results on a test of intellectual function performed about 1 year after the parent reports (mean age = 9.59, *SD* = 0.95), and an academic achievement score from the School registry when the participants attended high school (age 16–19 years, *n* = 104). To make the sample similar to the BGALS, 11 participants with an IQ score below 70 were excluded, together with one child defined as an outlier (>3 SD) according to casewise statistics. In total, 92 participants are included in the final BCS sample for this report. According to the Kiddie-SADS interview in the clinical phase of the first study wave, diagnosis was confirmed in 27 of the children (ADHD *n* = 9; anxiety *n* = 10; depression/dysthymia *n* = 2; OCD *n* = 1; Tourette syndrome/chronic tics *n* = 1; enuresis/encopresis *n* = 4).

The Regional Centre for Child and Youth Mental Health and Child Welfare, Uni Research, collaborated with Hordaland County Council to conduct the study. The BCS was approved by the Regional Committee for Medical and Health Research Ethics (REC), Western Norway. Parents gave written consent for participation when the children were below 16 years, and the adolescents consented themselves to participate when they were above 16 in accordance with Norwegian regulations.

### Measures

#### Parent Ratings of Inattention

Parents completed the nine relevant items from SNAP-IV (Swanson, 1992) in both the BGALS and BCS. The items cover the symptoms defined in the inattentive-disorganized dimension of ADHD, as described in the Diagnostic and Statistical Manual of Mental Disorders (DSM-IV, American Psychiatric Association, 2000). SNAP-IV uses four levels to evaluate each item, and these levels were included in the BGALS questionnaires. Parents rated each item with a value of 0 (“not at all”), 1 (“a little”), 2 (“pretty much”), or 3 (“very much”). We calculated a sum score (range = 0 to 27) for current analyses. The BCS used a three-level item Likert-type scale (0 = “not true,” 1 = “somewhat true,” or 2 = “certainly true”), consistent with the response metric of the remaining scales included in Wave 1 of the BCS. Thus, sum scores ranged from 0 to 18.

#### Intellectual Functioning

Full-scale IQ (FSIQ) was calculated from the WISC-III, scored according to American norms in the BGALS study (Wechsler, 1991) and according to Swedish norms for the BCS (Sonnander et al., 1998). The WISC-III is an extensively used measure of general cognitive abilities for children and adolescents, possessing good psychometric properties (Kaufman, 1994). A trained graduate student or test technician administered the full WISC-III to all participants. The WISC-III was administrated at the same time as the parent reports of inattention in the BGALS, when the children were 6–12 years old, and about 1 year after the parent reports in BCS, when the children were 8–10 years old.

#### Academic Achievement

The Wechsler Individual Achievement Test (WIAT; Wechsler, 1992) was included in the third wave of the BGALS (10 years after baseline assessment, Wave 1). The WIAT is a psychometrically sound assessment of academic achievement, with both internal consistency and test–retest reliability estimates above 0.85 for most composite scores (Wechsler, 1992). The Basic Reading and Math Reasoning scores, summed together, are included as a measure of academic functioning at follow-up.

For the BCS, academic achievement scores were provided by official registers from the Hordaland County for those who consented to the school registry linkage. In Norway, grades are assigned on a scale ranging from 1 to 6, with 6 being the highest grade (outstanding competence). A score of 2 (low level of competence) is the lowest passing grade, and 1 signifies a fail. The scores represent the average of all grades except for gym class. The achievement scores from both BGALS and BCS are calculated as continuous measures.

#### Covariates

In the BGALS sample, several important background variables were included as covariates: (a) mother’s highest-level education, (b) total household income, (c) ethnicity, and (d) age at baseline, all of which were ascertained via a demographic questionnaire at baseline. In the BCS, covariates were restricted to age at WISC-III testing and biological sex. Among families who answered the question about mother’s education (73.1%), all but two reported at least 12 years of education. Furthermore, all but one of the participants had at least one parent born in Norway, confirming the homogeneous ethnicity of the Norwegian population. Maternal education and ethnicity were therefore not included as covariates when analyzing the BCS data.

### Data Analytic Plan

Data from both studies were analyzed (SPSS version 23/24) according to the same analytic plan, including covariates that were specific to characteristics of the two countries. Group differences (between the ADHD and comparison subgroups within the BGALS and between males and females in the BCS) were initially calculated. Hierarchical linear regression analyses with academic achievement as the late-adolescent/early adult outcome measure were computed to investigate the effect of parent-reported inattention during childhood. In all analyses, relevant demographic variables were included in the first step. We then added inattention in the next step to investigate its direct effect on academic achievement. Then, to probe whether an effect of inattention was retained when intellectual function was covaried, intellectual function was included in a second step after demographics, followed by parent reports of inattention in a final step. Standardized change scores were used to reflect the unique contribution from the variables included in the regression models.

## Results

### Berkeley Girls with ADHD Longitudinal Study

Results are presented in **Table 1**. At inclusion, the ADHD group obtained significantly higher parent rated inattention scores than the comparison group (*p* < 0.001, *d* = 4.13), as well as significantly lower scores for FSIQ (*p* < 0.001, *d* = 0.94). The WIAT composite of reading and math achievement obtained during Wave 3 was far lower in the ADHD than in the comparison group (*p* < 0.001, *d* = 1.12). There were no significant group differences for the variables of age, household income, and mother’s education, but the % Caucasian was significantly higher in the ADHD group (*p* < 0.05).

**Table 1:** Results in the BGALS and BCS sample.

	BGALS		BCS		
	ADHD (*n* = 139)	CON (*n* = 87)	ALL (*n* = 92)	BOYS (*n* = 66)	GIRLS (*n* = 26)
Age	9.64 (1.68)	9.44 (1.65)	9.59 (0.95)	9.69 (0.95)	9.35 (0.90)
SNAP-IV	21.08 (4.79)	3.86 (3.44)	4.15 (4.33)	5.05 (4.62)	1.88 (2.32)
WISC-III	99.65 (13.56)	111.95 (12.71)	95.66 (12.05)	94.86 (12.64)	97.69 (10.33)
^∗^Academic achievement	188.47 (27.54)	214.73 (18.35)	4.03 (0.79)	3.96 (0.81)	4.19 (0.72)

*BGALS: Age = when included; SNAP-IV = four response categories; Academic achievement = WIAT score wave 3. BCS: Age = when tested; SNAP-IV = three response categories. ^∗^For the BGALS sample we used WIAT scores and for the BCS sample we used the mean score from official registry Wave 4*.

Bivariate correlation analyses including the full sample showed statistically significant negative correlations between (a) parent reports of childhood inattention and concurrent FSIQ (*r* = -0.353, *p* < 0.001), (b) parent reports of childhood inattention and academic achievement at Wave 3 (*r* = -0.374, *p* < 0.001), and (c) childhood FSIQ and Wave 3 achievement (*r* = 0.687, *p* < 0.001). When considering the two subsamples separately, only the correlations between FSIQ and academic achievement were statistically significant, in both the ADHD (*r* = 0.623, *p* < 0.001) and comparison subsamples (*r* = 0.593, *p* < 0.001). The entire sample was therefore included in the regression analysis.

The regression analyses (see **Table 2**) revealed that parent-reported inattention at Wave 1 significantly predicted academic achievement scores in high school (*p* < 0.001), explaining 10.4% of the variance beyond the covariates (mother’s education, household income, ethnicity, and child age at baseline). Academic achievement was also significantly predicted by FSIQ (*p* < 0.001), explaining 36.9% of the variance beyond the covariates. When inattention was included in the final stage after entry of FSIQ, the contribution from inattention remained statistically significant (*p* < 0.05), bringing the overall explained variance of the model to 52.9%.

**Table 2:** Predicting academic achievement.

	BGALS			BCS		
Predictor	*β*	*R*^2^	Δ*R^2^*	*β*	*R*^2^	Δ*R^2^*
**Model 1:**						
Demographics		0.148	14.8%		0.025	2.4%
Inattention	-0.334^∗∗^	0.252	10.4%	-0.398^∗∗^	0.163	13.8%
**Model 2:**						
Demographics		0.149	14.9%		0.025	2.4%
Intellectual	0.639^∗∗^	0.517	36.9%	0.431^∗∗∗^	0.238	18.2%
Inattention	-0.119^∗^	0.529	1.2%	-0.263^∗^	0.309	7.1%

*Model 1: Academic achievement ∼ Demographics + Inattention score; Model 2: Academic achievement ∼ Demographics + Intellectual function + Inattention. ^∗∗∗^*p* < 0.001; ^∗∗^*p* < 0.01; ^∗^*p* < 0.05. *β* = standardized coefficient, Δ*R^2^ = unique* R^2^ change*.

### Bergen Child Study

**FIGURE 1:**
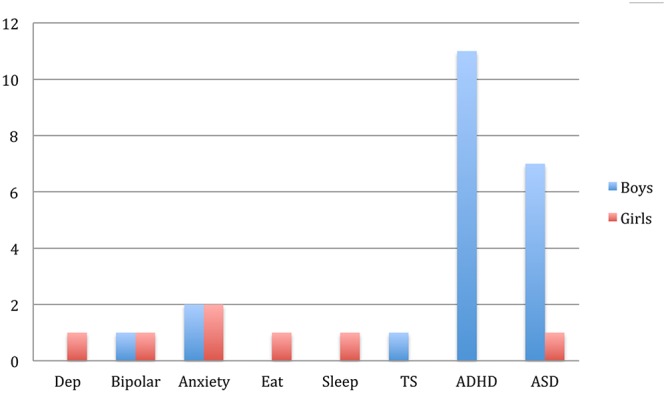
Number of self-reported psychiatric problems in high school (BCS). Dep, depression; Eat, eating disorder; Sleep, sleep disorder; TS, Tourette syndrome; ASD, Autism/Asperger syndrome.

The BCS sample included a higher number of boys (*n* = 66) than girls (*n* = 26). Boys and girls obtained similar results on all measures except for parent-reported inattention, which was significantly higher in boys than in girls (*p* < 0.001, *d* = 0.76) (see **Table 1**). There were 14 boys and 5 girls who reported at least one psychiatric disorder at the late adolescent data collection point. ADHD was reported in only boys (*n* = 11), all of whom had a comorbid disorder (see **Figure 1**). None of the participants had psychosis, intellectual disability, or known neurological disorders.

The correlations in the full sample were statistically significant for the relation between the childhood inattention and FSIQ scores (*r* = -0.273, *p* = 0.008), childhood inattention and the high-school academic achievement score (*r* = -0.402, *p* < 0.001), and childhood FSIQ and the high-school achievement score (*r* = 0.479, *p* = < 0.001). In a sex-specific correlation analysis, all correlations retained their statistical significance for boys (inattention/FSIQ, *r* = -0.293, *p* = 0.017; inattention/academic achievement *r* = -0.370*, p* = 0.002; FSIQ/academic achievement *r* = 0.532, *p* < 0.001), but for girls, only the correlation between inattention and academic achievement, *r* = -0.517, *p* = 0.007 remained significant. Sex was therefore kept as a covariate in the following regression analyses.

The linear regression analyses (see **Table 2**) revealed that parent reports of inattention significantly predicted academic achievement in high school (*p* = 0.001), explaining 13.8% of the variance beyond the two covariates (age and sex). Introducing intellectual function as a predictor explained 23.8% of academic achievement (*p* < 0.001), with 18.2% incremental variance explained by FSIQ. Inclusion of parent reports of inattention in the final step, following inclusion of the covariates and intellectual function, increased the explained variance to 30.9%, leaving the contribution from inattention statistically significant (*p* < 0.05).

Because the correlational pattern was different for boys and girls, the regression analysis was re-conducted separately for boys and girls with age as the only covariate. Inattention was still statistically significant for boys (*p* = 0.032) and girls (*p* = 0.008), while the effect of intellectual function was statistically significant only for boys (*p* < 0.001).

## Discussion

In the present study, including two culturally and diagnostically diverse samples, we found a significant and replicated link between parent reports of inattention in their primary school children and future academic achievement, approximately 10 years later. The independent contribution from parent-reported inattention was remarkably similar across the two study samples when sample-specific demographic variables were covaried. The impact of intellectual function on academic achievement was also strong in both samples, particularly so in BGALS and among boys in the BCS sample. Still, the effect of parent reports of childhood inattention was significant in both samples even when childhood intellectual function was taken into account.

As noted in the introduction, several studies have emphasized the impact of early inattentive behavior on future academic function, in both clinical samples of youth with ADHD (Lee and Hinshaw, 2006; Pingault et al., 2011; Garner et al., 2013) and community samples (Holmberg and Bolte, 2014; Gray et al., 2015). The present study extended these findings by including samples representing dimensions of ADHD symptoms, allowing the study of symptom severity above and beyond diagnostic categories *per se*. Our results showed that negative consequences associated with inattention were not restricted to children within the diagnostic category of ADHD. Moreover, there were similar magnitudes of correlation between both intellectual functioning and inattention with respect to later achievement in the ADHD and comparison samples for BGALS, confirming that also cognitive function has a strong influence on future academic achievement in children with ADHD as well as in youth in general (Miller and Hinshaw, 2010).

The extensive covariation of socio-economic factors in the BGALS was a necessary step, in order to demonstrate specificity of predictions to later achievement from childhood inattention and IQ. However, this level of statistical adjustment was not needed in the BCS because of the socio-economic homogeneity of the Norwegian society. The importance of taking cultural differences in socio-economic status into account is illustrated in studies of intellectual function. For example, the effect of socio-economic status on intellectual function is much weaker in samples from Nordic countries than in U.S. samples (Andersson et al., 1996; Osler et al., 2013; Ellertsen et al., 2016).

Even when adjusting for childhood intellectual functioning, the incremental prediction of later achievement from childhood inattention remained as an important predictor of later academic performance. In an educational contex, this replicated finding points to the importance of remediation and training procedures aimed at helping primary school children with inattention problems. Behavioral parental training, which has been proved successful in helping preschool children (Sonuga-Barke et al., 2013), may be of importance to parents to understand and help children with problems related to inattention. Similar training programs could be offered to primary school teachers—who also require classroom aides and other supports—focusing on the risk of negative social labeling that may accompany school-related problems. Cognitive training programs have recently been increasingly popular for school children with ADHD (see, e.g., Rapport et al., 2013; Tamm et al., 2017). These types of interventions address problems that are highly relevant for inattentive behavior, including what is commonly referred to as executive dysfunction (see Colomer et al., 2017). Despite the negative findings of the review of Cortese et al. (2015) for specific cognitive training regarding youth, programs with a more general targeting of congitive and emotional function may still be found to provide some lasting benefit.

### Strengths and Limitations

The inclusion of two diagnostically and culturally diverse samples, using similar methodological procedures, is a main strength of the present study. Such inclusion, however, also led to several limitations, affecting the strength of our conclusions. The selection of informant (i.e., parent reports) and the measures of cognitive function (i.e., performance on a test of intellectual function) and inattention were motivated largely by our objective of including parallel measures in both studies. Still, we believe that these selections have given us results of clinical importance. Parent reports are commonly the most available information about a child’s behavior when assessed in a clinical situation; the total score of the included questionnaire on inattention is well-validated; and the full-scale IQ score is commonly available as a general measure of cognitive function. Still, questions may be raised about the specificity of the parent reports, in that parents may find it difficult to contrast inattention with characteristics of other aspects of problematic behavior. Other limitations are related to critical differences in study designs, leading to somewhat different age groups and not quite overlapping time-lines in our longitudinal designs.

## Conclusion

Inattention is one of the hallmarks of ADHD. The present study shows that features of this behavior, in particular from a parent’s perspective, are of crucial importance in predicting high-school achievement about a decade later—an effect that is not restricted to children within the diagnostic category of ADHD. Furthermore, the time span between parent reports of inattention and assessment of academic achievement was longer than in most previous studies (∼10 years), suggesting that the effects of inattention in primary school may yield consequences into adult life. Inattention tends to lead to a cascade of other problems (Gillberg, 2010; Sonuga-Barke and Halperin, 2010), including peer-related problems (see Bellanti and Bierman, 2000; Andrade and Tannock, 2014) and mood disorders (Rajendran et al., 2013; Lundervold et al., 2016). As a result, remediation of problems related to inattention may provide benefit not only for academic and later vocational problems but also for social interactions and general mental health (see Richardson et al., 2012; Fried et al., 2016).

## Author Contributions

AL: data collection, design, statistical analysis and main responsibility for the manuscript; JM: data collection, design, statistical analysis and comments on the manuscript; MH: data collection, statistical analysis, comments on the manuscript; SH: data collection, design and main comments on the manuscript.

## Conflict of Interest Statement

The authors declare that the research was conducted in the absence of any commercial or financial relationships that could be construed as a potential conflict of interest.
